# A Multiscale Review of Behavioral Variation in Collective Foraging Behavior in Honey Bees

**DOI:** 10.3390/insects10110370

**Published:** 2019-10-25

**Authors:** Natalie J. Lemanski, Chelsea N. Cook, Brian H. Smith, Noa Pinter-Wollman

**Affiliations:** 1 Department of Ecology and Evolutionary Biology, University of California, Los Angeles, CA 90024, USA; nmpinter@ucla.edu; 2 School of Life Sciences, Arizona State University, Tempe, AZ 85287, USA; cncook1@asu.edu (C.N.C.); BrianHSmith@asu.edu (B.H.S.)

**Keywords:** *Apis mellifera*, collective behavior, foraging, honey bees, individual variation, learning

## Abstract

The emergence of collective behavior from local interactions is a widespread phenomenon in social groups. Previous models of collective behavior have largely overlooked the impact of variation among individuals within the group on collective dynamics. Honey bees *(Apis mellifera)* provide an excellent model system for exploring the role of individual differences in collective behavior due to their high levels of individual variation and experimental tractability. In this review, we explore the causes and consequences of individual variation in behavior for honey bee foraging across multiple scales of organization. We summarize what is currently known about the genetic, developmental, and neurophysiological causes of individual differences in learning and memory among honey bees, as well as the consequences of this variation for collective foraging behavior and colony fitness. We conclude with suggesting promising future directions for exploration of the genetic and physiological underpinnings of individual differences in behavior in this model system.

## 1. Introduction

Collective behavior emerges from individual-based local rules without central control. Traditional models of collective behavior have considered all individuals in a group as identical agents that follow the same behavioral rules [1,2]. However, individuals differ from one another due to both internal and external causes [3,4]. Individuals comprising a group may be of different ages and sexes, and they can vary in their gene expression, developmental pathways, neurological activity, and hormonal regulation [5,6]. Furthermore, individuals differ in their responses to external stimuli, such as interactions with other group members and with the biotic and abiotic environment. This variation among individuals can have important influences on the emergence of collective behavior [7,8,9,10,11,12], for example, because certain individuals can have a disproportionate impact on collective outcomes [13,14].

Social insects are ideal model systems to examine the impact of individual variation on collective outcomes. In social insects, there is variation among individuals relating to which task each individual tends to perform (e.g., foraging vs. brood care) [8], as well as the way in which they perform tasks [15]. Because natural selection acts on the colony rather than on the individual agents [16], the fitness consequences of this variation depend on how it affects the collective behavior. Thus, studying social insects allows a multilevel approach that integrates the role of individual variation, from the underlying mechanism of variation among individuals (such as genetics and physiology) to the collective, ecological, and evolutionary consequences [17].

The honey bee, in particular, has been studied for decades at many levels of organization [1,18,19,20]. Thus, there is a strong foundation from which to integrate the causes and consequences of variation from the individual to the collective levels. Honey bees form elaborate societies that perform a variety of functions, including brood care, hive construction, and foraging. Here, we focus on the causes and consequences of individual variation to collective foraging.

Collective foraging in honey bees is performed by a subset of workers [21]. Some workers forage for nectar, while others forage for pollen [22]. Some bees leave the hive early in the day to scout for new resources, while other bees leave the hive in response to recruitment by the scouts or other foragers [23]. In addition to these broad patterns of variation, there is also variation within each type of forager (scouts or recruits), in how they perform these tasks, and in how they learn about their environment [24,25,26]. In the context of foraging, learning plays an important role because honey bees need to identify suitable resources, return to them repeatedly, and communicate the location and quality of the resources to other colony members.

In this paper, we review the genetic, physiological, and learning mechanisms underlying individual variation in foraging behavior of honey bees. We detail the ecological and evolutionary consequences of this variation. We end the paper with suggestions for promising future directions of investigation that both emerge from our review of the literature and can advance the study of the role of individual variation in shaping the collective foraging of bees.

## 2. Variation in Individual Learning and Behavior

In honey bees, learning plays an important role in foraging. Foragers must learn to associate relevant stimuli, such as color or odor, with a reward, such as nectar or pollen. If individuals vary in the way they make this association or in how they respond to the association, the colony will have a variety of ways to integrate information. Floral resources change many times over the foraging lifetime of a honey bee, with changes occurring within and across days and weeks [27,28]. Thus, a forager needs to be prepared to quickly learn about the best resources and then change its preference when a better resource becomes available.

The study of learning and memory in the honey bee has a long tradition [20]. The pioneering work of Karl von Frisch [29] over 100 years ago showed that honey bees have color vision by training them to respond differentially to different colors. In the past 100 years, many studies have revealed nonassociative, associative, and operant mechanisms of learning and memory in honey bees [30,31,32,33,34,35]. All of these learning mechanisms need to be integrated into a honey bee forager’s decision-making process as it collects pollen, nectar, and water [36].

Comparatively little attention has been paid to how individual foragers differ in the way they learn and make decisions about resources. A possible reason for this oversight is that many protocols for studying learning are not equipped to identify these differences. In particular, assays that involve freely flying foragers attract individuals that are motivated to learn the particular contingencies associated with reward [37,38,39]. Individuals that are not predisposed to learning the specific rewards will not be responsive to the experimental situation. Nevertheless, substantial individual variation has been found in multiple types of learning in honey bees, and this variation has important consequences for collective behavior.

One type of variation in learning that exists in honey bees is differential sensitivity to not receiving reinforcement [40]. This form of nonassociative learning is called latent inhibition (LI) [41,42,43]. Exposure to an odor without reinforcement causes some honey bees (high latent inhibition) to learn it slowly, or not at all, when that odor is subsequently paired with reinforcement in a way that normally produces a robust conditioned response [40]. In contrast, other honey bees (low latent inhibition) learn to associate odors with reward regardless of previous exposure.

Honey bees have also been found to vary in their reversal learning abilities [44,45]. In reversal learning, individuals are trained to discriminate an odor associated with sucrose reinforcement (A+) from a different odor associated with nothing (X_0_), and then tested on their ability to learn the opposite association when the reinforcement contingency is reversed (A_0_/X+) [44]. Individuals that are high in latent inhibition tend to also be poor at reversal learning, suggesting these learning behaviors have a shared genetic or neurological basis [44].

Foragers vary in their responsiveness to sucrose, the main unconditioned stimulus involved in honey bee learning. While some individuals require a high concentration sucrose reward to produce a conditioned stimulus response, other individuals will respond to low sucrose concentrations or even water alone [22,46]. Variation in sucrose responsiveness is important for foraging because it influences which food foragers collect. Foragers show preferences for collecting pollen, nectar, or water for thermoregulation [22]. Foragers with low sucrose response thresholds tend to forage for water, while foragers with high thresholds tend to forage for nectar; foragers with an intermediate threshold typically forage for pollen [22,47].

## 3. Genetic Mechanisms

Within a honey bee colony, there is high genetic variation among workers due to the queen mating with up to 20 drones (haploid males) [18,48]. Because the queen uses sperm from many or all of these drones to fertilize the hundreds of eggs she lays every day, colonies contain multiple “patrilines” of workers that vary genetically due to different haploid fathers and recombination of the queen’s chromosomes during meiosis [49,50]. As a result, workers exhibit natural heritable variation. To elucidate the effect of genetics on colony function, Smith and others have experimentally manipulated colony genetics by instrumentally inseminating queens with a single drone to produce workers with an average of 75% relatedness [51].

Within-colony genetic variation affects how different workers learn. Several studies have shown that the individual differences in multiple types of learning are heritable in honey bees. Using the Cape honey bee, which reproduces, in part, parthenogenetically, Brandes and Menzel [52] showed that selection for differences in olfactory conditioning performance reveal differences in visual conditioning performance in freely flying bees. As there was no correlated response in sensitivity to sucrose, they suggested that the selection was on a central neural mechanism involved in learning ability, as opposed to differences in sensitivity to the sensory stimuli used for conditioning [53]. Brandes further proposed that additive genetic factors contribute to these individual differences in learning and memory performance [54,55].

More recently, Smith and colleagues demonstrated the heritability of two different forms of learning: latent inhibition and reversal learning. Similar to workers, queens and drones exhibit variation in LI. By testing the LI of drones and virgin queens, then inseminating queens with sperm from drones that showed like learning performance, they demonstrated single generation selection responses in worker progeny, which tended to exhibit similar LI to their parents [44]. Moreover, there was a correlated selection response between LI and reversal learning [40,44], suggesting that there might be pleiotropy or linkage disequilibrium between the genetic factors that underlie these two learning traits. Using quantitative trait locus mapping, Chandra and colleagues [56] identified a region of the honey bee genome that has a strong association with LI, which they called LRN1. One gene in that region encodes a tyramine receptor (*amtyr1* for *Apis mellifera* tyramine receptor 1), suggesting that this biogenic amine could play a role in this type of learning. However, there are several other genes in that region that could be involved as well [56].

Genetic variation also influences the tendency of foragers to act as scouts. Different patrilines within a colony may differ in their likelihood of scouting [57]. In addition, certain honey bees tend to specialize in scouting across multiple situations, including nest site selection and searching for food [58]. In both situations, scouts have similar gene expression in their brains [59], which is distinct from non-scout individuals [58]. Specifically, scout bees show differences in expression of several important neurotransmitters, including dopamine, octopamine, and gamma-Aminobutyric acid (GABA) [58]. Cook and colleagues [24] found that in addition to having elevated tyramine levels, scouts tend to be higher in LI than recruits, suggesting that genetic differences in LI may be one mechanism underlying variation in scouting behavior. Foragers with high latent inhibition may be more prone to seek novel resources once known resources begin to be depleted. In contrast, foragers with low latent inhibition may be more likely to continue to exploit known sites, even as they become depleted.

The differences in the type of food, nectar or pollen, that foragers collect are driven by genetic differences. Page and colleagues [60] used artificial selection to demonstrate heritable variation in pollen collection. By rearing queens from colonies that store either high or low amounts of pollen, over several generations they produced a “high pollen hoarding” strain that packs the colony with pollen, and a “low pollen hoarding” strain that packs the colony with nectar [60,61]. Later studies demonstrated that three quantitative trait loci, PLN1, PLN2, and PLN3, explain most of the variation in the pollen versus nectar phenotypes [62,63]. Interestingly, PLN2 [62] is in the same region of the genome as LRN1 [56], suggesting that a gene or gene(s) at this locus (e.g., *amtyr1*) play a role in learning, as well as in preferences for nectar vs. pollen. The PLN1, PLN2, and PLN3 loci are related to several important neurotransmitter and hormonal signaling pathways, including tyramine, octopamine, and vitellogenin [64]. These pathways, thus, might interact to influence several aspects of individual behavior, including how large of a pollen load a bee collects, how it responds to sucrose, how it learns, and at what age it begins to forage [64]. However, the mechanism for how the neurotransmitter or hormone receptor genes in the region of the LRN1 or PLN2 loci influence such a diverse set of behaviors is not yet understood.

Though genetic variation influences worker behavioral specialization, workers retain a large degree of behavioral plasticity, allowing them to respond to changes in colony needs. This plasticity is partly driven by the effects of social environment on gene expression. For instance, honey bee foragers produce a pheromone that inhibits foraging in younger nestmates and removal of foragers alters gene expression in young bees, leading to earlier onset of foraging behavior [65,66]. Exposure to queen mandibular pheromone also represses the expression of several genes involved in foraging [67]. One gene overexpressed in foragers is the *Amfor* gene, which codes for a cGMP-dependent protein kinase (PKG); treating bees with an artificial analog of this gene’s product induces precocious foraging in young bees, similar to the effect of forager removal [68]. The decision to collect pollen or nectar is also influenced by the colony’s changing nutritional needs. For example, when a colony is rearing a brood, more protein is needed. Brood pheromone, given off by developing larvae, induces hormonal changes in individuals that stimulate pollen collection [69,70]. In contrast, the presence of stored pollen, assessed by trophallactic transfers of protein from nurse bees, inhibits pollen collection by foragers [71,72].

## 4. Fitness Consequences of Collective Behavior

Collecting food for the entire colony is a major challenge for honey bees. Honey bees from the same colony forage across areas spanning up to several hundred square kilometers, and at linear distances as far as 9 km from the hive [73]. To increase efficiency, foraging labor is divided into different task groups. Foragers specialize in the type of food they collect, as well as whether they scout for new resources or are recruited to known resources. Variation in learning among individuals in the colony is important for this division of labor because learning influences which of the foraging tasks individuals are most likely to perform, as detailed above [22,24].

The collective decisions that result from differences in behavior at the individual level have an important impact on a colony’s foraging success, and therefore on its fitness. Both the range of behavioral variation and the frequency of each behavioral type in the colony can impact collective outcomes [9]. A wider range of behavioral types can increase the likelihood that complementary behavioral types, such as scouts and recruits, will be represented in the colony. For instance, Mattila and Seeley [57] found that greater genetic diversity increases the likelihood that the rare scout-like genotypes are present in a colony, thus increasing a colony’s ability to discover new resource patches.

Collective dynamics can also be determined by the frequency of each behavioral type. When foraging, colonies face a trade-off between exploiting known resources and exploring for new ones. This trade-off can be resolved by allocating the task of scouting only to a sufficient number of individuals to allow locating new resources without losing too many foragers to the costs of exploration. There is an optimal ratio of scouts to recruits, or explorers to exploiters, which maximizes collective foraging [74]. However, this balance may change as a function of the structure of the landscape in which the colony forages [75,76,77]. For example, theoretical models have established that when resources are concentrated into a small number of highly rewarding patches, colonies perform best with few scouts and many recruits [23,78]. In contrast, when resource patches are small, evenly distributed, and easy to locate, successful colonies invest more in scouting than in recruitment [23,78]. Furthermore, having many recruits is beneficial when patches are highly variable in quality, because recruitment drives foragers to the highest quality patches [79,80]. Empirical tests have provided support for the prediction that high investment in recruitment is most beneficial when resources are distributed in few large patches and are variable in quality [28,75].

The behavioral composition of the colony has consequences for collective pollen foraging, which can have major impacts on worker health and larval development [81,82]. For instance, genetic differences in the sucrose response thresholds of individual foragers scale up to influence the total amount of pollen that the colony collects [60,61]. Because pollen is often variable in nutritional quality [81], having fewer pollen scouts and more recruits allows colonies to focus efforts on the highest quality pollen resources [76,83].

## 5. Conclusions and Open Questions

The work we have reviewed suggests many new lines of research across multiple scales that span genes, neural networks, individual behavior, and collective function. In fact, the case could be made that the honey bee is now poised to be one of the most important animal models for substantial inroads into an integrated, multilevel understanding of social control of collective decisions.

### 5.1. Neurophysiological Mechanisms

Individual and collective behaviors are driven by proximate physiological mechanisms. Several neurophysiological and hormonal mechanisms have been identified as being important for honey bee learning and foraging behavior, including tyramine, octopamine, GABA, and vitellogenin. Tyramine, in particular, seems to play a role in both foraging and learning behaviors, as it differs between scouts and recruits [24]. In addition, scout bees have significantly higher tyramine and octopamine in their brains compared to recruited bees [24]. Using electrophysiology to measure neuronal responses, low LI foragers show a decreased net neuronal response in their antennal lobes to a familiar odor compared to high LI foragers [84]. Additional research focused on gene expression, such as RNAseq, could help clarify the role of these neurochemicals and their receptors in both learning and foraging behavior.

### 5.2. Forward and Reverse Genetics

Several studies reviewed above have productively used reverse genetics such as quantitative trait locus (QTL) mapping to identify genetic loci and gene networks that influence individual variation in foraging behavior. One gene of particular interest that has been identified in independent QTL-based screens is *amtyr1*, which is found in the PLN2/LRN1 area of the genome [56,62]. The genes coding for octopamine, dopamine, and GABA receptors are other likely candidates, as expression of these genes differs between scouts and non-scouts, and scouting behavior is modulated by agonists and antagonists of these neurochemicals [58]. Further work needs to be done in establishing other loci in the honey bee genome that have effects on behavior, and how epigenetic factors come into play among already identified loci and any others that come up in new screens.

Important next steps would be to employ forward genetics to manipulate the expression of genes. This approach could be accomplished by using gene editing technology, such as CRISPR (clustered regularly interspaced short palindromic repeats) [85], or transcription disrupting techniques, such as RNA interference (RNAi) [86,87]. These techniques could be used to dissect the causal roles that genes play in shaping collective behavior through their impact on the behavior of individuals.

### 5.3. Concluding Remarks

Variation among individuals can have major consequences for the emergence of collective behavior in social groups. Future work exploring the genetic, developmental, and physiological causes of this variation will be an important contribution toward a more complete understanding of the evolution of collective behavior. Honey bees have so far proven to be an excellent model system for elucidating the genetic underpinnings of variation in learning and its consequences for collective foraging behavior. Future work on this model system is likely to prove fruitful for furthering our understanding of the causes and consequences of individual variation across multiple scales.
